# Chromosome 3 Anomalies Investigated by Genome Wide SNP Analysis of Benign, Low Malignant Potential and Low Grade Ovarian Serous Tumours

**DOI:** 10.1371/journal.pone.0028250

**Published:** 2011-12-06

**Authors:** Ashley H. Birch, Suzanna L. Arcand, Kathleen K. Oros, Kurosh Rahimi, A. Kevin Watters, Diane Provencher, Celia M. Greenwood, Anne-Marie Mes-Masson, Patricia N. Tonin

**Affiliations:** 1 Department of Human Genetics, McGill University, Montreal, Canada; 2 The Research Institute of the McGill University Health Centre, Montreal, Canada; 3 Division of Clinical Epidemiology and Segal Cancer Centre, Lady Davis Research Institute, Jewish General Hospital, Montreal, Canada; 4 Department of Pathology, Centre Hospitalier de l'Université de Montréal (CHUM), Montréal, Canada; 5 Department of Pathology, McGill University and McGill University Health Centre (MUHC), Montréal, Canada; 6 Centre de recherche du Centre hospitalier de l'Université de Montréal (CRCHUM), Institut du cancer de Montréal, Montreal, Canada; 7 Division of Gynecologic Oncology, Université de Montréal, Montreal, Canada; 8 Department of Oncology, McGill University, Montreal, Canada; 9 Department of Epidemiology, Biostatistics and Occupational Health, McGill University, Montreal, Canada; 10 Department of Medicine, Université de Montréal, Montreal, Canada; 11 Department of Medicine, McGill University, Montreal, Canada; Univesity of Texas Southwestern Medical Center at Dallas, United States of America

## Abstract

Ovarian carcinomas exhibit extensive heterogeneity, and their etiology remains unknown. Histological and genetic evidence has led to the proposal that low grade ovarian serous carcinomas (LGOSC) have a different etiology than high grade carcinomas (HGOSC), arising from serous tumours of low malignant potential (LMP). Common regions of chromosome (chr) 3 loss have been observed in all types of serous ovarian tumours, including benign, suggesting that these regions contain genes important in the development of all ovarian serous carcinomas. A high-density genome-wide genotyping bead array technology, which assayed >600,000 markers, was applied to a panel of serous benign and LMP tumours and a small set of LGOSC, to characterize somatic events associated with the most indolent forms of ovarian disease. The genomic patterns inferred were related to *TP53*, *KRAS* and *BRAF* mutations. An increasing frequency of genomic anomalies was observed with pathology of disease: 3/22 (13.6%) benign cases, 40/53 (75.5%) LMP cases and 10/11 (90.9%) LGOSC cases. Low frequencies of chr3 anomalies occurred in all tumour types. Runs of homozygosity were most commonly observed on chr3, with the 3p12-p11 candidate tumour suppressor region the most frequently homozygous region in the genome. An LMP harboured a homozygous deletion on chr6 which created a *GOPC-ROS1* fusion gene, previously reported as oncogenic in other cancer types. Somatic *TP53*, *KRAS* and *BRAF* mutations were not observed in benign tumours. *KRAS*-mutation positive LMP cases displayed significantly more chromosomal aberrations than *BRAF*-mutation positive or *KRAS* and *BRAF* mutation negative cases. Gain of 12p, which harbours the *KRAS* gene, was particularly evident. A pathology review reclassified all *TP53*-mutation positive LGOSC cases, some of which acquired a HGOSC status. Taken together, our results support the view that LGOSC could arise from serous benign and LMP tumours, but does not exclude the possibility that HGOSC may derive from LMP tumours.

## Introduction

Epithelial-stromal tumours of the serous histopathological subtype represent the largest group of epithelial ovarian cancers (EOC) and account for significant morbidity and mortality. Ovarian serous tumours may present as benign, low malignant potential (LMP) or malignant disease. Benign tumours account for up to 60% of ovarian serous tumours, present bilaterally in 20% of cases, and are cured through surgical removal of the disease [1]. LMP tumours account for up to 15% of ovarian serous tumours and present bilaterally in 30% of cases. Although about 75% of LMP tumours are stage I at diagnosis, where survival rates exceed 90% [1], [2], patients with advanced stage disease may die from complications due to extragonadal spread throughout the pelvic cavity. Approximately 15% of LMP tumours may recur up to 20 or more years after the initial diagnosis, and these cases usually have a poor outcome [1], [3]. About 30% of all ovarian serous tumours are malignant and 60% of these cases are bilateral [1]. Serous tumours make up more than 50% of all malignant EOC. Although various grading methods have been used [4], it appears that the vast majority of malignant serous tumours are high grade ovarian serous carcinomas (HGOSC), with only about 10% presenting as low grade carcinomas (LGOSC). Treatments for both include surgery and chemotherapy, but most cases are diagnosed at advanced stages where the overall 5-year survival rate is less than 30%. Although patients with LGOSCs have a longer survival than those with HGOSCs, they respond poorly to conventional platinum and taxane-based chemotherapy, suggesting that the molecular pathways involved in the etiology of the diseases may differ [5]. Although approximately 10% of EOC, particularly tumours of the serous subtype, occur in women harbouring germline mutations of the cancer susceptibility genes *BRCA1* or *BRCA2* [6], the etiology of the remainder of ovarian serous neoplasms remains unknown.

Karyotyping and array comparative genomic hybridization (aCGH) studies of benign, LMP and malignant serous tumours indicate an increasing frequency of chromosomal abnormalities, with the most extensive aneuploidy and structural abnormalities occurring in malignant tumours [7]–[11]. Genetic analyses of *TP53* have identified rare somatic mutations in benign, LMP tumours and LGOSCs, and a very high frequency in HGOSCs [12]. Mutually exclusive somatic mutations in either *KRAS* or *BRAF* are often reported in LMP tumours and LGOSCs (30–50%), but rarely in HGOSCs (<12%) [13], [14]. This mutation spectrum has been used as an argument that favours at least two distinct, but not mutually exclusive, pathways for the development and progression of ovarian serous tumours. One pathway involves a continuum of development involving benign, LMP tumours and LGOSCs, originating from surface epithelial cells of the ovary. The other pathway describes the *de novo* development of HGOSCs originating from either ovarian surface epithelial cells, or epithelial cells of the fallopian tube fimbriae [4].

Defining the genes involved in the etiology of ovarian serous neoplasms would provide a means to further stratify patients for optimal treatment regimens, as well as identify new molecular pathways to explore in the development of biomarkers. This is particularly prescient for LMP cases given that the majority of patients do not succumb to the disease, although most cases are usually subjected to aggressive management. Although studies of DNA ploidy in LMP tumours have been used to stratify patients for aggressive treatment, the overall impact on survival is not clear [15]. Karyotype studies have implicated chromosome 3p genes in EOC [16], [17], and loss of heterozygosity (LOH) analyses have suggested that 3p genes may function as tumour suppressors [18]–[21]. We have previously reported LOH of 3p14-pcen in benign, LMP tumours, LGOSCs and HGOSCs [19], [21]. Although the studies were limited by sample size, it is tempting to speculate that gene(s) residing in this genomic region may be involved in the tumourigenesis of ovarian serous neoplasms. This notion is supported by functional complementation studies involving the transfer of ‘normal’ 3p fragments, including the 3p12-pcen region, which rendered an aggressive EOC cell line harbouring LOH of the 3p arm, non-tumourigenic [22], [23]. LOH of 3p25-ptel was also reported in benign, LGOSCs, and HGOSCs [19], [24], suggesting more than one tumour suppressor gene may be involved in the etiology of ovarian serous neoplasms. Whole genome expression analyses and targeted analyses of 3p25-ptel and 3p14-pcen genes also have identified promising candidates for further molecular analyses [23]–[25].

In this study we have performed an extensive genetic analysis of benign and LMP ovarian serous tumours to further characterize somatic genetic events associated with the most indolent form of ovarian disease. We performed a targeted LOH analysis of the 3p12-pcen locus of interest generated from our previous analyses of benign, LMP and malignant ovarian carcinomas [19], in benign ovarian serous tumours to determine the extent of loss of 3p alleles in this disease. To further characterize genomic anomalies, we applied high-density genome-wide genotyping bead array technology to benign and LMP ovarian serous tumour samples. Genome-wide genotyping array studies have already shown the occurrence of specific anomalies, such as 3p loss, attesting to earlier findings that genomic aberrations are not necessarily random in malignant EOC (reviewed in Gorringe *et al.*, 2009 [26]). However, genotyping array analyses have largely focused on HGOSCs [27], and previous genome-wide studies of benign, LMP tumours and LGOSCs were limited by the density of genetic markers or by sample size [7]–[11], [28]–[31]. We relate our results to the mutational spectra derived from *TP53*, *KRAS* and *BRAF* genetic analyses, as these genes are mutated in ovarian tumours with varying frequencies depending on the pathology of the disease. In some cases, we were also able to investigate synchronous bilateral ovarian tumours. We also analyzed a set of LGOSC, as these cancer samples have rarely been genetically characterized due to their paucity relative to HGOSC cases. This study represents the largest sample of ovarian serous tumours examined to date using high density genotyping technologies. The integration of targeted genetic analyses with global genomic effects may contribute to our understanding of the etiology of benign and LMP ovarian serous tumour samples. The results of our targeted genetic and genomic analyses support the hypothesis that LGOSC could arise from serous benign and LMP tumours, but do not exclude the possibility that HGOSC may also be derived from LMP tumours.

## Results

### Genetic analysis of chromosome 3p

LOH of 3p has been reported in up to 20% of benign ovarian serous tumours [19], [20]. As previous studies were limited by sample size, we used polymorphic microsatellite repeat markers to investigate LOH of regions on 3p in 50 benign ovarian tumour samples. We focused our analysis on the 3p26.2, 3p21.31, 3p12.3, 3p12.2, and 3p11.2 regions shown to exhibit LOH in serous benign and/or malignant tumours [18]–[20]. Although the analysis was informative for at least one marker per region examined in 78–90% of the samples, no evidence of LOH was observed in any of the samples analyzed.

**Figure 1:**
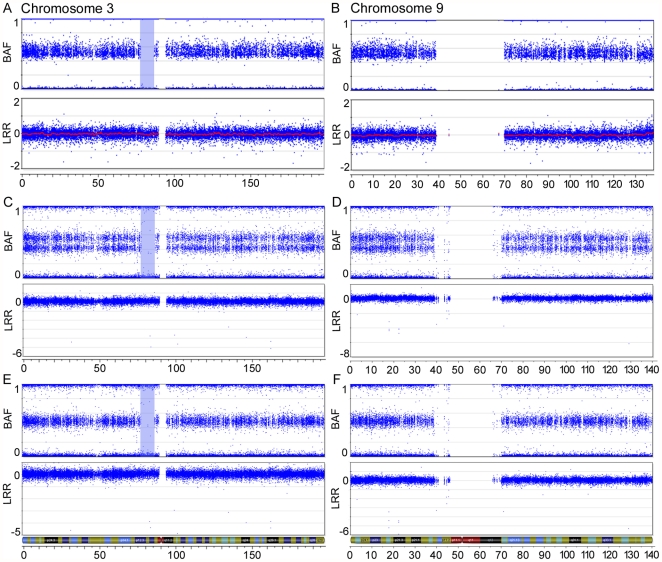
SNP array imaging results for chr3 and chr9 of the benign serous tumour 1781T. SNP array imaging results for chr3 (**A, C, E**) and chr9 (**B, D, F**) of the benign serous tumour 1781T, using Illumina's HumanHap300-Duo Genotyping BeadChip (**A** and **B**) and Illumina's Human610-Quad Genotyping BeadChip (**C–F**). Two different DNA preparations were used with the HumanHap610-Quad Genotyping BeadChip. The top plot of each figure shows the B allele frequency (BAF) for each SNP marker aligned to its chromosomal position. In heterozygous diploid cells, alleles are present in AA, AB or BB pairs. The B Allele frequencies for these possible allele pairs are 0, 0.5 or 1, respectively. Any deviation from this ratio indicates a chromosomal aberration. In one DNA preparation, the double row in the BAF plot indicates allelic imbalance of SNP markers across the entire chromosome (**C** and **D**). A 9.1 Mb ROH is observed on chr3 and is highlighted in blue. No markers are located in the centromeric region of either chromosome, as noted by a lack of markers in both the B allele frequency and Log R ratio (LRR) plots. The bottom plot of each figure contains the Log R ratio, which provides an indication of the copy number for each SNP marker aligned to its chromosomal position. Note the absence of a drop in the Log R ratio in the highlighted ROH.

To increase the resolution of markers in order to detect LOH events in tumour samples, we applied Illumina's HumanHap300-Duo Genotyping BeadChip, which assays approximately 317,500 SNPs across the human genome, to three benign ovarian tumour samples. As proof of principle, we investigated sample 1781T, a benign ovarian serous tumour that has been shown to exhibit LOH of 3p14-pcen. Samples BOV-1329GT and BOV-2564DT, which did not exhibit evidence of LOH in the present study, were also examined. BeadChip analysis identified a 9.1 Mb run of homozygosity (ROH) at 3p12-p11 in 1781T that did not display a corresponding decrease in the Log R ratio, which would have been consistent with a deletion occurring in this region (**Figure 1A**). No 3p anomalies were inferred from the BeadChip analyses of samples BOV-1329GT and BOV-2564DT (data not shown). Genetic analysis of DNA from normal tissues from case sample 1781T using seven polymorphic microsatellite repeat markers suggested that the 3p ROH also occurred in constitutional DNA (data not shown). A higher density array, the Human610-Quad Genotyping BeadChip (610K), which contains over 600,000 markers, was used to genotype DNA extracted from two portions of the 1781T tumour specimen. Interestingly, 1781T-A exhibited consistent allelic imbalance across the entire lengths of chr3 and chr9, in contrast to normal genotypes observed on all chromosomes in the 1781T-B DNA preparation (**Figure 1**).The 3p12-p11 ROH is present in both preparations, affirming earlier findings that this ROH is likely present in constitutional DNA. These results are interesting in light of our recent studies that suggest the presence of an ovarian cancer tumour suppressor gene located in the 3p12-pcen region [22], [23].

### High-density genome-wide genotyping of benign tumours, LMP tumours and LGOSCs

To investigate the possibility that LOH analyses underestimated the frequency of 3p abnormalities in benign and LMP serous tumours, we applied the 610K BeadChip technology to an additional 21 benign ovarian serous cases (32 tumours) and 53 LMP ovarian serous cases (58 tumours), of which 10 benign and 5 LMP cases included samples taken from both the left and the right ovaries. We also included 11 LGOSC cases (12 tumours), for which both bilateral tumours of one patient were arrayed. HGOSCs have already been shown to demonstrate LOH and abnormalities of 3p using genotyping arrays [26], [27].

**Figure 2:**
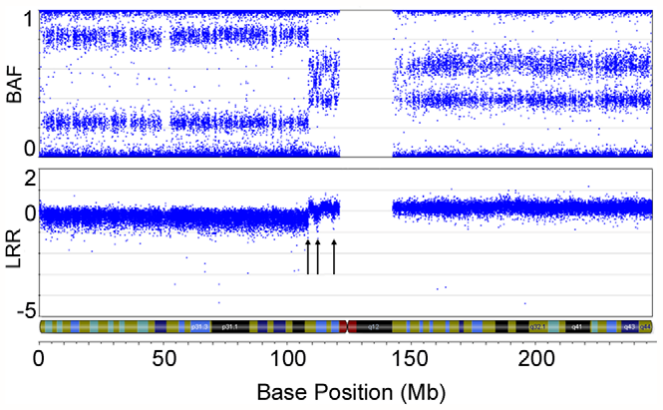
Example of intrachromosomal breaks and allelic imbalance in an LMP tumour. SNP array imaging results for chr1 of LMP sample TOV-845T. Several intrachromosomal breaks are denoted by arrows on 1p, and are visualized by breaks in the continuity of both the B allele frequency and Log R ratio plots. Note the Log R ratio indicates loss of copy number for most of the 1p arm, with gains of copy number near the centromere. The double row in the BAF plot observed on 1q indicates allelic imbalance of SNP markers across the entire chromosomal arm. Note the Log R ratio for the 1q arm averages above 0, indicating a gain of copy number.

Using the Genome Viewer module of the BeadStudio software, we visually assessed the data, which was aligned according to genomic position. The B allele frequency and Log R ratio were examined in order to infer allelic imbalance of whole chromosomes or chromosomal arms and intrachromosomal breaks (**Figure 2** **;** **Tables 1** **,** **2** **,** **3**). As summarized in **Table 2**, allelic imbalance of 3p was observed in only two LMP samples, TOV-1068T and TOV-3922GT, both of which also harboured allelic imbalances of other chromosomes. Breaks involving the 3p arm were observed in two LMP tumours, TOV-942T and TOV-1685T. Chromosome 3p breaks were more frequently observed in LGOSCs (4 of 11 cases); however, intrachromosomal breaks were also observed on other chromosomes in all of these cases (**Table 3**). Overall, chromosomal aberrations were more commonly observed in LMP cases (30 of 53 cases or 56.6%) than in benign cases (3 of 22 cases, 13.6%) (**Tables 1** **and** **2**). The most commonly affected chromosomal arms in LMP cases were 12p (12/53), 12q (9/53), 8p (7/53), 8q (7/53), 1p (6/53), and 22q (6/53). Allelic imbalance was more frequently observed on chr12 and chr8, whereas intrachromosomal breaks were observed more often on 1p and 22q. Chromosomal abnormalities were observed in all but two of the LGOSC samples (TOV-682T and TOV1284T).

**Table 1:** Chromosomal aberrations and mutations observed in benign ovarian serous tumours.

Sample			Pathology	Age	Stage	Imbalance of whole chromosome or chromosomal arms	Intrachromosomal breaks	*KRAS*	*BRAF*	*TP53*
BOV-392	DT	GT	Benign	57	-	-	-	-	-	-
BOV-846	DT	GT	Benign	67	-	-	-	-	-	-
BOV-1172	DT	GT	Benign	66	-	-	-	-	-	-
BOV-1588	DT	GT	Benign	56	-	-	-	-	-	-
BOV-2314	DT	GT	Benign	64	-	-	-	-	-	-
BOV-2889	DT	GT	Benign	65	-	-	-	-	-	-
BOV-3057	DT	GT	Benign	52	-	-	-	-	-	-
BOV-3097	DT	GT	Benign	73	-	-	-	-	-	-
BOV-3150	DT	GT	Benign	56	-	-	-	-	-	-
BOV-3268	DT	GT	Benign	48	-	-	-	-	-	-
BOV-1329	GT		Benign	26	-	-	-	-	-	-
BOV-2564	DT		Benign	53	-	-	-	-	-	-
1781	T		Benign	66	-	3,9	-	-	-	-
BOV-1207	DT		Benign	51	-	-	13q	-	-	-
BOV-1296	DT		Benign	71	-	-	-	-	-	-
BOV-1332	DT		Benign	67	-	-	-	-	-	-
BOV-1761	GT		Benign	63	-	-	-	-	-	-
BOV-2023	DT		Benign	70	-	-	21q	-	-	-
BOV-2328	DT		Benign	52	-	-	-	-	-	-
BOV-2331	GT		Benign	57	-	-	-	-	-	-
BOV-2418	GT		Benign	71	-	-	-	-	-	-
BOV-2506	DT		Benign	67	-	-	-	-	-	-

Description of chromosomal aberrations and mutations observed in a panel of 32 benign ovarian serous tumours from 22 patients. All chromosomal arms which display an intrachromosomal break or allelic imbalance are shown for each tumour, along with the corresponding mutations. T, tumour; DT, tumour on right ovary; GT, tumour on left ovary; EPT, tumour on omentum.

**Table 2:** Chromosomal aberrations and mutations observed in LMP ovarian serous tumours.

Sample			Initial Pathology	Revised Pathology	Age	Stage	Imbalance of whole chromosome or chromosomal arms	Intrachromosomal breaks	*KRAS*	*BRAF*	*TP53*	Other
TOV-335	DT		LMP		44	IIIB	5, 8, 12, 15, 20	-	c.35G>T; p.Gly12Val	-	-	
TOV-929(B)	T		LMP		54	IIIA	12, 18	-	c.35G>T; p.Gly12Val	-	-	
TOV-978	T		LMP		34	IA	8, 12, 20	6p, 13q	c.35G>A; p.Gly12Asp	-	-	
TOV-1068	T		LMP		58	IIIC	3,5, 7, 8, 12, 18	-	c.35G>A; p.Gly12Asp	-	-	
TOV-1215	GT		LMP		27	IIIA	12p, 19	1p, 6q, 22q	c.34G>C; p.Gly12Arg	-	-	
TOV-2262	DT		LMP		62	IB	1q, 8, 12	1p	c.35G>T; p.Gly12Val	-	-	
TOV-3922	GT		LMP		65	IA	2, 3, 6, 7, 9, 12, 20	-	c.35G>A; p.Gly12Asp	-	-	
TOV-1228	GT		LMP		42	IIIC	-	7p, 16q	c.35G>T; p.Gly12Val	-	-	
TOV-4105	GT		LMP		66	IA	-	8q, 12p	c.35G>T; p.Gly12Val	-	-	
TOV-3492	DT		LMP		41	IIC	-	-	c.35G>T; p.Gly12Val	-	-	
TOV-3882	DT		LMP		49	IIIA	-	-	c.35G>A; p.Gly12Asp	-	-	
N3426	DT		LMP		60		-	-	c.35G>T; p.Gly12Val	-	-	
TOV-1010	DT		LMP		31	IB	7, 8	21q	-	c.1799T>A; p.Val600Glu	-	
TOV-1010		GT	LMP		31	IB	-	-	-	-	-	
TOV-4269	DT		LMP		21		7p	7q	-	c.1799T>A; p.Val600Glu	-	
TOV-696	GT		LMP		41	IIIC	-	12p, 15q, 17q	-	c.1799T>A; p.Val600Glu	-	
TOV-920	DT	GT	LMP		45	IIIA	-	3q, 22q	-	c.1799T>A; p.Val600Glu	-	
TOV-991	DT		LMP		67	IB	-	-	-	c.1397G>T; p.Gly466Val	-	
TOV-991		GT	LMP		67	IB	-	11p	-	c.1397G>T; p.Gly466Val	-	
TOV-2173	T		LMP		49	IA	-	7q	-	c.1799T>A; p.Val600Glu	-	
TOV-3165	GT		LMP		35	IA	-	2p	-	c.1799T>A; p.Val600Glu	-	
TOV-933	DT		LMP		77	IA	-	-	-	c.1799T>A; p.Val600Glu	-	
TOV-984	DT		LMP		34	IB	-	-	-	c.1799T>A; p.Val600Glu	-	
TOV-1267	DT		LMP		44	IIIA	-	-	-	c.1799T>A; p.Val600Glu	-	
TOV-1300	GT		LMP		47	IIIC	-	-	-	c.1799T>A; p.Val600Glu	-	
TOV-3094	GT		LMP		29	IIIC	-	-	-	c.1799T>A; p.Val600Glu	-	
TOV-3973	GT		LMP		62	IA	-	-	-	c.1799T>A; p.Val600Glu	-	
TOV-4262	GT		LMP		26	IIIC	-	-	-	c.1799T>A; p.Val600Glu	-	
TOV-1685	GT		LMP	LMP	26	IA	5p, 13q, 17p	1p, 1q, 2p, 2q, 3p, 3q, 4p, 4q, 5q, 6p, 6q, 7p, 7q, 8p, 8q, 9p, 9q, 10q, 14q, 16p, 17q, 18q, 19p, 21q, 22q, Xp, Xq	-	-	c.1024delC; p.Arg342na; putative stop aa344	
TOV-942	GT		LMP	LMP	58	IC	8p, 17p, 20q, X	3p, 4p, 6p, 9p, 9q, 10q, 12p, 12q, 13q, 17q, 19p, 20p, 22q	-	-	-	*KRAS* amplification
TOV-4054	DT		LMP		50	IC	-	6q	-	-	-	*GOPC-ROS1* fusion protein
TOV-4054		GT	LMP		50	IC	11, 12	6q	-	-	-	*GOPC-ROS*1 fusion protein
TOV-206	DT		LMP		28	IIIC	12	-	-	-	-	
TOV-845	T		LMP		50	IIIC	1q	1p	-	-	-	
TOV-1694	DT		LMP		71	IIIA	17q	-	-	-	-	
TOV-1775	DT	GT	LMP		49	IIIA	1q, 16	-	-	-	-	
TOV-3028	GT		LMP		74	IA	16q	1p	-	-	-	
TOV-306	DT		LMP		62	IIIC	-	15q, 22q	-	-	-	
N945	T		LMP		64	IIB	-	6q	-	-	-	
TOV-1101	GT		LMP		52	IIIC	-	19q	-	-	-	
TOV-1319(A)	T		LMP		80	IA	-	22q	-	-	-	
TOV-2395	DT		LMP		33	IA	-	2q	-	-	-	
TOV-3546	DT		LMP		51	IIIC	-	1p	-	-	-	
TOV-107	GT		LMP		63	IA	-	-	-	-	-	
TOV-838	GT		LMP		38	IB	-	-	-	-	-	
TOV-916	T		LMP		65	IIIA	-	-	-	-	-	
TOV-1112	T		LMP		69		-	-	-	-	-	
TOV-1157	T		LMP		61	IA	-	-	-	-	-	
TOV-1533	GT		LMP		60	IA	-	-	-	-	-	
TOV-1607	T		LMP		58	IB	-	-	-	-	-	
TOV-1615	DT		LMP		62	IA	-	-	-	-	-	
TOV-1915	T		LMP		61	IA	-	-	-	-	-	
TOV-2005	DT		LMP		54	I	-	-	-	-	-	
TOV-2563	DT		LMP		50	IB	-	-	-	-	-	
TOV-3423	DT		LMP		40	IIB	-	-	-	-	-	
TOV-3703	GT		LMP		69	IA	-	-	-	-	-	

Description of chromosomal aberrations and mutations observed in a panel of 58 LMP serous tumours from 53 patients. Two tumours displaying a high level of chromosomal instability were reevaluated by a gynecologic pathologist (TOV-1685GT and TOV-942GT). All chromosomal arms which display an intrachromosomal break or allelic imbalance are shown for each tumour, along with the corresponding mutations. T, tumour; DT, tumour on right ovary; GT, tumour on left ovary; EPT, tumour on omentum.

**Table 3:** Chromosomal aberrations and mutations observed in LGOSC.

Sample			Initial Pathology	Revised Pathology	Age	Stage	Imbalance of whole chromosome or chromosomal arms	Intrachromosomal breaks	*KRAS*	*BRAF*	*TP53*
832	T		LGOSC		37	IIIC	-	1p, 2q, 5q, 6p, 6q, 19q	c.35G>A; p.Gly12Asp	-	-
682	T		LGOSC		40	IIIC	-	-	-	c.1799T>A; p.Val600Glu	-
TOV-854	DT	GT	LGOSC		61	IIIC	4, 5, 7, 8, 11, 13, 15, 20, X	-	-	c.1799T>A; p.Val600Glu	-
TOV-947	DT		LGOSC	LMP	53	IA	4, 14, 17p, 18p, 21, X	1p, 1q, 2p, 2q, 3p, 3q, 5q, 6p, 6q, 7p, 7q, 8q, 9p, 9q, 10q, 11p, 12q, 13q, 15q, 16q, 17q, 18q, 19p, 19q, 20q, 22q	-	-	c.725G>A; p.Cys242Tyr
TOV-553	EPT		LGOSC	HGSOC	48	IIIC	16p, 17q	1p, 1q, 2p, 2q, 3p, 3q, 4p, 4q, 5p, 5q, 6p, 6q, 7p, 7q, 8p, 8q, 9p, 9q, 10p, 10q, 11p, 11q, 12p, 12q, 13q, 14q, 15q, 16q, 17p, 18p, 18q, 19p, 19q, 20p, 20q, 21q, 22q, Xp, Xq	-	-	c.659A>G; p.Tyr220Cys
TOV-81	DT		LGOSC	non invasive implant	66	IIIC	17p, 18p, Xq	1p, 1q, 2p, 2q, 3p, 3q, 4p, 4q, 5p, 5q, 6p, 6q, 7p, 7q, 8p, 8q, 9p, 9q, 10p, 10q, 11p, 11q, 12p, 12q, 13q, 14q, 15q, 16p, 16q, 17q, 18q, 19p, 20p, 20q, 22q, Xp	-	-	c.818G>A; p.Arg273His
TOV-490	T		LGOSC	HGSOC	71	IIIC	1q, 2q, 3q, 4, 5p, 5q, 6p, 7p, 9q, 10p, 14, 16p, 16q, 17, 18q, 20,	1p, 2p, 3p, 6q, 7q, 9p, 10q, 12p, 12q, 13q, 18p, 19p, 19q, 21q, 22q, Xp, Xq	-	-	c.382delC; p.Pro128na; putative stop aa169
TOV-812	EPT		LGOSC	Metastatic serous	70	IIIC	6q, 9, 16, 17, 18, 22q, X	1p, 1q, 4q, 11p, 19p	-	-	c.455_456insC; p.Pro152na; putative stop aa180
635	T		LGOSC		48	IIIC	1p, 1q, 4, 9, 22q, X	8p, 17p	-	-	-
TOV-1284	T		LGOSC		39	IIIC	-	-	-	-	-
TOV-1949	T		LGOSC		52	IIIC	8q	3q, 5p, 7p	-	-	-

Description of chromosomal aberrations and mutations observed in a panel of 12 LGOSC samples from 11 patients. Five tumours displaying a high level of chromosomal instability and *TP53* mutations were reevaluated by a gynecologic pathologist (TOV-947DT, TOV-553EPT, TOV-81DT, TOV-490T and TOV-812EPT). All chromosomal arms which display an intrachromosomal break or allelic imbalance are shown for each tumour, along with the corresponding mutations. T, tumour; DT, tumour on right ovary; GT, tumour on left ovary; EPT, tumour on omentum.

Bilateral tumours from 16 samples in this study were genotyped. None of the 10 paired bilateral benign tumours exhibited any evidence of genomic anomalies. Of the five paired LMP samples examined, one or both tumour samples exhibited evidence of chromosomal abnormalities. Some of these cases exhibited identical (cases TOV-1775 and TOV-920) or similar (case TOV-4054) abnormalities, suggesting the possibility of common clonal origins in these cases, as has been proposed for malignant ovarian cancers [32]. An identical spectrum of chromosomal abnormalities was observed in the one case of paired LGOSC samples (case TOV-854).

Homozygous deletions may be inferred by identifying markers associated with a downward deviation of the Log R ratio and the absence of allele frequency scores. Null alleles resulting from somatic homozygous deletions are of particular interest, as they may affect the function of tumour suppressor genes. Furthermore, breaks occurring adjacent to cancer-associated genes may affect their regulation. **Table S2** provides the coordinates where both alleles are likely to be deleted, along with the affected genes. Some of the intervals are known to harbour germline copy number variants (CNV) as reported in the Database of Genomic Variants (projects.tcag.ca/variation). Thirty-six homozygous deletions were found to be unique to a single case. Some of these deletions may possibly affect the function of genes located within or adjacent to the deleted intervals (**Table 4**). Homozygous deletions were observed in benign, LMP tumours and LGOSCs, although relative to the number of samples in each group, homozygous deletions were observed more often in LGOSCs. This is likely to be an overestimate, as LGOSC sample TOV-490T harboured several chromosomes with reduced copy number. On these chromosomes the Log R ratio is decreased, resulting in the coincidental appearance of three adjacent markers with a Log R ratio of ≥−2.

**Table 4:** Homozygous deletions observed in a single sample.

Chr	Sample	Pathology	Flanking SNPs	Genomic Location	Cytoband	Maximum Size	# Markers within Deletion	Nearest Upstream Gene	Genes included in deletion	Full Gene Name	Nearest Downstream Gene	High Grade Amplifications	Homozygous Deletions
**2**	BOV-2506DT	Benign	rs1983365;rs1175861	4,198,741–4,224,068	2p25.3	25,328 bp	6/7	*BC043283/BC062482*	*-*	-	*AK307324*	2	22
**2**	TOV-933DT	LMP	rs869817;rs17546105	12,016,999–12,031,541	2p25.1	14,543 bp	4	LPIN1*	*-*	-	*AK001558*	3	0
**2**	BOV-1172DT/GT	Benign	rs17864582;rs1028145	51,922,567–51,928,042	2p16.3	5,476 bp	8	*NRXN1*	*AK127244*	mRNA AK127244	*ASB3*	1	0
**2**	TOV-916T	LMP	rs1011572;rs12328023	53,551,600–53,561,575	2p16.2	9,976 bp	3	*NRXN1*	*-*	-	*ASB3*	3	0
**2**	TOV-845T	LMP	rs12615852;rs12469535	242,974,521–243,044,147	2q37.3	69,627 bp	8	*C2ORF85*	***LOC728323***	hypothetical LOC728323	Telomere	0	8
**2**									***AK125674***	mRNA AK125674			
**4**	BOV-3097DT/GT	Benign	rs28625386;rs7693378	70,323,237–70,636,709	4q13.2	104,491 bp	6/7	*UGT2B11*	***UGT2B28***	UDP glucuronosyltransferase 2 family, polypeptide B28	*UGT2B4*	0	44
**4**	TOV-854 DT/GT	LGSOC	rs1507939;rs950206	116,165,372–116,180,555	4q26	15,184 bp	4	*NDST4*	*-*	-	*MIR1973*	1	2
**4**	TOV1112T	LMP	rs10518388;rs2013332	122,280,335–122,291,522	4q27	11,188 bp	5	*TNIP3*	*QRFPR*	pyroglutamylated RFamide peptide receptor	ANXA5*	0	10
**4**	BOV-1588DT/GT	Benign	rs7692005;rs4692824	171,253,046–171,288,473	4q33	35,428 bp	3	*AADAT*	*-*	-	*HSP90AA6P*	0	0
**5**	TOV-306DT	LMP	rs4374757;rs4571472	15,719,350–15,721,360	5p15.1	2,011 bp	4	*ANKH*	*FBXL7*	F-box and leucine-rich repeat protein 7	*MARCH11*	13	6
**5**	BOV-3057DT/GT	Benign	rs4704943;rs12187915	155,470,446–155,500,631	5q33.2	30,186 bp	3	*KIF4B*	*SGCD*	sarcoglycan, delta	*PPP1R2P3*	0	3
**6**	TOV-3165GT	LMP	rs9350099;rs926274	19,039,776–19,051,695	6p22.3	11,920 bp	4	*AK098665*	*-*	-	*AK097585*	2	0
**6**	TOV-306DT	LMP	rs9469655;rs2495975	33,935,903–33,944,014	6p21.31	8,112 bp	4/5	*AL832447*	*-*	-	*MIR1275*	1	0
**6**	TOV-1949T	LGSOC	rs2749135;rs11155845	101,491,425–101,507,345	6q16.3	15,921 bp	3	*ASCC3*	*-*	-	*GRIK2*	0	0
**6**	TOV4054DT	LMP	rs492132;rs12194183	117,643,433–117,885,959	6q22.1	242,527 bp	70/74	*VGLL2*	***ROS1***	c-ros oncogene 1, receptor tyrosine kinase	*NUS1*	0	1
**6**									***DCBLD1***	discoidin, CUB and LCCL domain containing 1			
**6**									***GOPC***	golgi-associated PDZ and coiled-coil motif containing			
**6**	TOV-490T	LGSOC	rs9374781;rs606955	119,444,784–119,457,914	6q22.31	13,131 bp	3	*MCM9*	*FAM184A*	family with sequence similarity 184, member A	MAN1A1*	1	0
**6**	TOV-490T	LGSOC	rs6900527;rs7761698	149,437,584–149,442,245	6q25.1	4,662 bp	3	*UST*	*-*	-	*TAB2*	0	0
**7**	BOV2328DT	Benign	rs10085387;rs11763921	97,389,030–97,404,277	7q21.3	15,248 bp	3	*TAC1*	*-*	-	*ASNS*	6	4
**9**	TOV-490T	LGSOC	rs10739110;rs7865244	6,697,128–6,715,730	9p24.1	18,603 bp	3	*GLDC*	*-*	-	*KDM4C*	4	0
**9**	TOV-490T	LGSOC	rs10960291;rs10511574	11,827,014–11,951,204	9p23	124,191 bp	38/39	*PTPRD*	*-*	-	*TYRP1*	5	10
**9**	TOV-490T	LGSOC	rs8181148;rs4977974	25,282,007–25,294,701	9p21.3	12,695 bp	3	*ELAVL2*	*-*	-	*TUSC1*	3	28
**9**	635T	LGSOC	rs4149303;rs2065412	107,594,515–107,598,740	9q31.1	4,226 bp	3	*LOC286367*	***ABCA1***	ATP-binding cassette, sub-family A (ABC1), member 1	*SLC44A1*	1	0
**9**	TOV4262GT	LMP	rs11788366;rs12237388	119,262,628–119,287,555	9q33.1	24,928 bp	3	*PAPPA*	*ASTN2*	astrotactin 2	*TRIM32*	0	0
**12**	TOV-553EPT	LGSOC	rs7971309;rs1863552	108,423,276–108,459,275	12q23.3	36,000 bp	3	*LOC728739*	*-*	-	*WSCD2*	1	0
**13**	BOV-2314DT/GT	Benign	rs7998352;rs1928393	34,134,809–34,149,388	13q13.2	14,580 bp	3	*KL*	*STARD13*	StAR-related lipid transfer (START) domain containing 13	*RFC3*	2	0
**13**	TOV-490T	LGSOC	rs1198316;rs1211304	50,370,205–50,381,016	13q14.2	10,812 bp	3	*KPNA3*	*-*	-	*CTAGE10P*	2	1
**13**	TOV-1228GT	LMP	rs9546330;rs2669264	83,787,475–83,793,975	13q31.1	6,501 BP		*SPRY2*	*-*	-	*SLITRK1*	3	1
**13**	BOV-392DT/GT	Benign	rs7987913;rs9517112	98,527,866–98,536,863	13q32.2	8,998 bp	4	*RAP2A*	*-*	-	*IPO5*	5	0
**16**	TOV4262GT	LMP	rs183112;rs1861320	55,527,682–55,541,040	16q12.2	13,359 bp	3	*IRX6*	***MMP2***	Matrix metalloproteinase 2	*LPCAT2*	0	0
**17**	TOV-490T	LGSOC	rs8075188;rs917344	69,387,158–69,399,273	17q24.3	12,116 bp	3	*KCNJ2*	*-*	-	***SOX9***	3	0
**18**	TOV4262GT	LMP	rs644016;rs11662635	55,164,547–55,170,274	18q21.31	5,728 bp	3	*ONECUT2*	*-*	-	*FECH*	0	0
**19**	BOV-3150DT/GT	Benign	rs6511105;rs7254995	20,612,645–20,728,777	19p12	116,133 bp	15	*ZNF826P*	***AF338193***	mRNA AF338193	*ZNF626*	2	19
**19**									***ZNF737***	zinc finger protein 737			
**19**	TOV-696GT	LMP	rs12110;rs4805110	35,660,508–35,669,071	19q13.12	8,564 bp	3	*FXYD7*	***FXYD5***	FXYD domain containing ion transport regulator 5	*FAM187B*	3	0
**20**	BOV-3150GT	Benign	cnvi0010506;rs3969184	26,248,774–28,118,678	20p11.1-q11.1	1,869,905 bp	5/6	centromere	*-*	-	*FRG1B*	1	0
**22**	TOV-490T	LGSOC	rs5748755;rs4819923	17,426,401–17,433,210	22q11.1	6,810 bp	3	*HSFY1P1*	*-*	-	*GAB4*	1	0
**X**	TOV-490T	LGSOC	rs5963931;rs5918139	41,064,184–41,116,530	Xp11.4	52,347 bp	3	*LOC100132831*	***USP9X***	ubiquitin specific peptidase 9, X-linked	*DDX3X*	0	2

Deletions are mapped on the Human Feb. 2009 (GRCh37/hg19) assembly of the human genome, except for the deletions flanked by non-SNP markers, which are mapped to the Human March 2006 (NCBI36/hg18) assembly. Flanking SNPs refers to the SNP markers flanking the homozygously deleted SNPs, and represent the largest possible size of the deletion. The genes located within and directly upstream and downstream from the hypothesized deleted regions are indicated. The genes that contain exons which may fall in the region of deletion are bolded. Genes found to be differentially expressed in Bonome *et al.*, 2005 are indicated by an asterisk (*). High grade amplifications and homozygous deletions refer to regions identified by the Sanger Cancer Genome Project.

Given the large ROH overlapping the 3p12-p11 region in the benign tumour sample 1781T, we investigated whether ROHs of this interval were also observed in other samples. This analysis was restricted to the benign and LMP samples, as they exhibited low levels of generalized genomic instability. We examined ROHs larger than 5 Mb, as previous studies have shown that smaller ROHs, particularly those less than 1.5 Mb, may be common occurrences [33], [34].There were no significant differences in the occurrence of at least one ROH >5 Mb per sample studied: 4/22 (18.1%) benign cases and 11/53 (20.4%) LMP cases contained at least one (**Table 5**). Notable is the large number of ROHs (n = 14) observed in the benign bilateral tumour samples BOV-1588DT and BOV-1588GT, as compared with benign and LMP cases exhibiting no (n = 61), one (n = 8), two (n = 5) or four (n = 1) ROHs >5 Mb. Both the left and the right ovarian tumours exhibited the same pattern of ROHs, accounting for about 7% of the genome. Genotyping of peripheral blood DNA from the same patient suggested that the ROHs occur in the germline and were not somatically acquired during the development of these tumours (data not shown). Interestingly, more ROHs were observed on chr3 than on any other chromosome (**Table 5**). Two LMP samples (TOV-1694DT and TOV-933DT) exhibited ROHs overlapping the 3p12-p11 ROH observed in the benign sample 1781T. Additionally, two benign and/or LMP tumour samples displayed overlapping ROHs located at 2.6–5.1 Mb and 190.2–196.4 Mb on chr3.

**Table 5:** ROHs longer than 5 Mb observed in benign and LMP tumours.

Sample			Pathlogy	Chr	Location (MB)	Size (bp)	# SNPs in region
BOV-1588	DT	GT	Benign	1	160.7–194.5	33,841,667	6654
				1	196.2–205	8,752,119	2007
				2	5.0–23.0	18,066,407	4085
				2	235.5–242.7	7,160,696	1656
				3	128.9–173.1	44,169,552	8290
				7	145.8–157.6	11,483,243	2810
				11	89.1–94.6	5,530,828	1005
				12	51.8–62.6	10,847,129	1827
				13	24.6–43.6	18,960,867	4658
				15	29.2–34.3	5,117,368	1316
				21	18.7–27.5	8,753,964	2144
				22	14.4–36.3	21,832,954	5490
				22	41.2–49.6	8,361,420	2645
				X	19.4–29.4	10,007,381	1267
BOV-1172	DT	GT	Benign	1	155.7–161.8	6,055,835	1491
BOV-2506	DT		Benign	13	49.3–60.9	11,593,401	2076
N1781	T		Benign	3	78.4–87.4	9,078,581	1206
N3426-RT	DT		LMP	3	190.2–196.4	6,176,699	1173
				6	39.0–82.4	43,394,086	8120
				10	12.7–25.6	12,881,505	3541
				X	48.0–68.4	20,413,402	1608
TOV-206	DT		LMP	1	37.9–55.6	17,698,088	2995
				15	57.6–66.7	9,077,633	2004
TOV-916	T		LMP	2	169.9–206.4	36,453,242	6293
				3	190.0–196.7	7,768,872	1594
TOV-1694	DT		LMP	3	71.7–128.3	56,621,515	9502
				15	40.1–51.5	11,391,842	2027
TOV-3882	DT		LMP	3	2.6–8.4	5,795,529	1963
				3	65.3–71.7	6,417,920	1437
TOV-4105(A)	GT		LMP	3	0–5.1	5,082,955	1947
				3	177.1–186.6	9,530,536	1788
TOV-1267	DT		LMP	X	71.9–77.6	5,692,402	314
TOV-107	GT		LMP	X	55.1–67.3	12,153,847	538
TOV-1775	DT	GT	LMP	8	24.5–29.7	5,229,945	1429
TOV-3165	GT		LMP	17	32.3–48.1	15,814,993	2498
TOV-933	DT		LMP	3	77.2–101.8	24,659,990	3046

# SNPs in the region refers to the number of polymorphic SNP markers located within the ROH. Only benign and LMP cases were examined in this analysis.

### Genetic analysis of *TP53*, *BRAF* and *KRAS* and association with genomic anomalies

Mutations of *KRAS*, *BRAF* and *TP53* were only detected in LMP tumours and LGOSCs (**Table 2**). As reported in independent studies, samples with mutations in *KRAS* or *BRAF* were mutually exclusive. Concordant mutation results were observed in all but one of the bilateral tumour samples (LMP case TOV-1010DT/GT). There were significantly more *KRAS* and *BRAF* mutations (26 of 53, 49.1%) and fewer *TP53* mutations (1 of 53, 1.9%) in LMP cases as compared with *KRAS* and *BRAF* mutations (3 of 11, 18.1%) and *TP53* mutations (5 of 11, 45.5%) in LGOSCs (**Tables 2** **and** **3**) (p = 0.00049).

**Figure 3:**
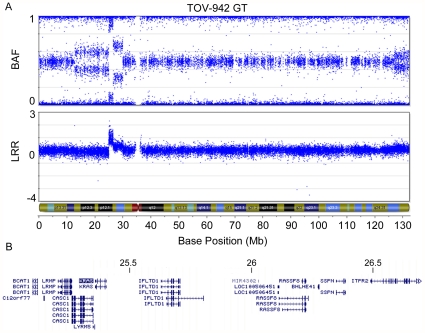
High level amplification of a 1.59 Mb region containing *KRAS* in an LMP sample. SNP array imaging results for chr12 of LMP sample TOV-942GT. A high-grade amplification of a discrete 1.59 Mb region (arrow) containing the proto-oncogene *KRAS* is observed (**A**). Depiction of the amplified region that contains 12 genes, including *KRAS* (arrow) (**B**).

In general, the LMP and LGOSC cases with somatic *TP53* mutations harboured disorganized genomes, particularly large numbers of intrachromosomal breaks (**Tables 2** **and** **3**). The LMP sample with a *TP53* mutation (TOV-1685GT) has 30 of 41 chromosomal arms harbouring an aberration, similar to the average number (33.4) of chromosomal arms harbouring an aberration in the *TP53* mutation positive LGOSCs. LMP cases with *KRAS* mutations contained an average of 5.3 chromosomal arms harbouring an aberration, whereas cases with *BRAF* mutations had an average of 1 chromosomal arm with an aberration. LMP mutation-negative tumours had an average of 1.5 chromosomal arms with an aberration. In the LMP tumours, there were significantly more *KRAS* mutation-positive cases that were associated with a gain of 12p (8 of 12, 66.7%) than there were in *KRAS* mutation-negative tumours (4 of 41, 9.8%) (p = 0. 0.0002). This is an interesting observation, as *KRAS* is located at 12p12.1. Moreover, the only other LMP sample to exhibit overt disorganization of its genome, sample TOV-942GT, harboured a high-level 1.59 Mb amplification containing 12 genes, including the *KRAS* locus (**Figure 3**).

A gynecologic pathologist independently reviewed the LMP and LGOSC samples that were found to harbor *TP53* mutations in a blinded manner to confirm their histopathological classification. All LMP samples retained their classification status. Interestingly, none of the LGOSC samples harbouring *TP53* mutations maintained their designation. TOV-553EPT and TOV-490T were reclassified as high grade carcinomas; TOV-812EPT was reclassified as a metastatic serous carcinoma, grade not determined; TOV-947DT was reclassified as a possible LMP; and TOV-81DT was reclassified as a non-invasive implant (**Tables 2** **and** **3**).

### Global analysis of copy number aberrations of benign, LMP and LGOSCs

Genotyping data were analyzed by GenoCNA to evaluate various states of copy number variations that include allelic content occurring within each group of benign and LMP samples. The LGOSCs were not analyzed, given the small number of cases within the group and the fact that a number of cases were later designated by histopathology as not LGOSC.

**Figure 4:**
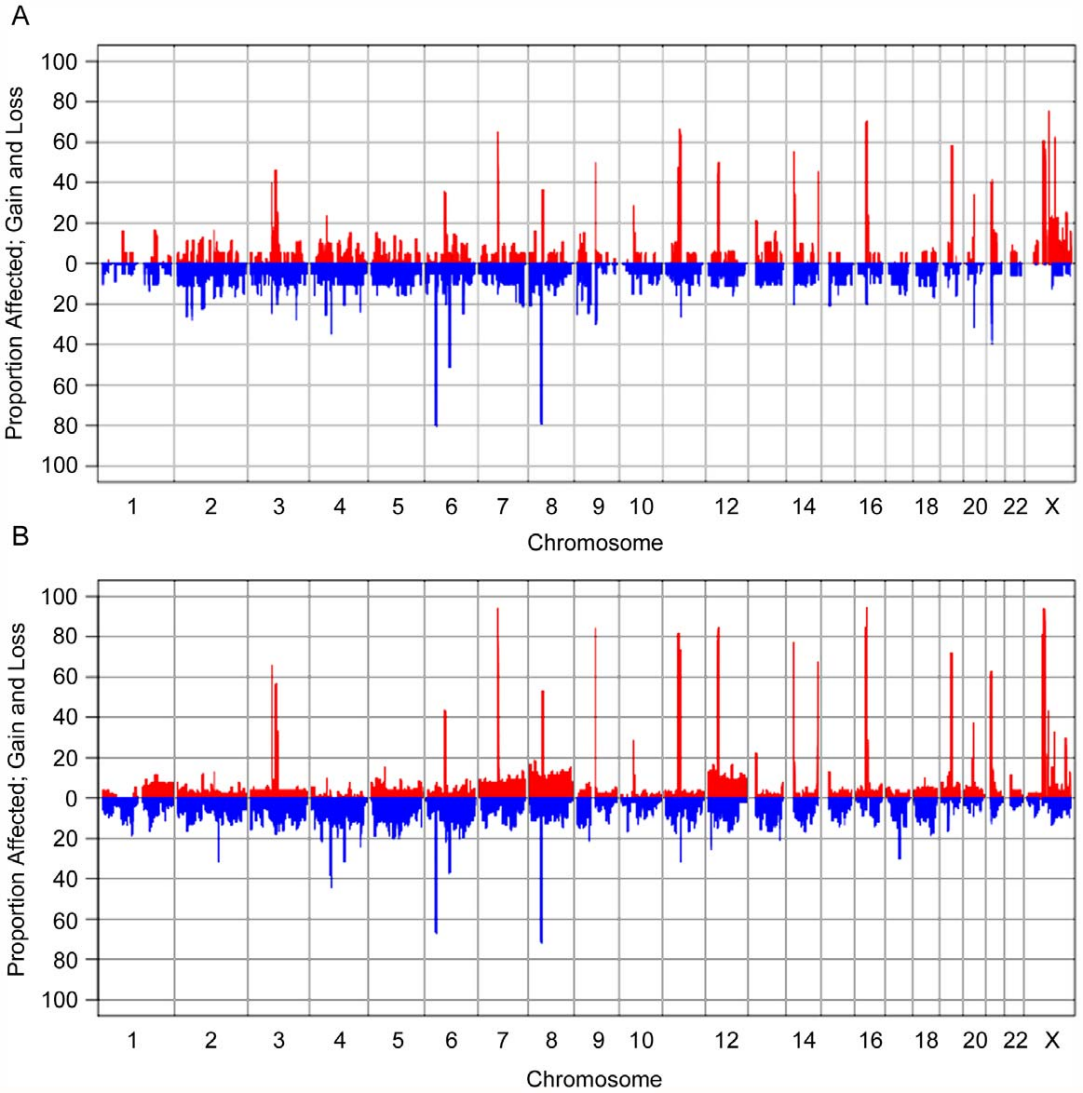
GenoCNA graphs showing gain and loss in serous benign tumour samples and serous LMP samples. GenoCNA graphs showing gain (red) and loss (blue) in 20 serous benign tumour samples (**A**) and 53 serous LMP samples (**B**). Peaks describing gain in >30% of samples represent repetitive regions around centromeres and/or telomeres. Peaks describing loss in >30% of samples represent common CNVs that often display loss of copy number. Somatic gains and losses of chromosomes are visible in the GenoCNA graph of the LMP samples, such as loss of 1p and gain of 1q, gain of chr7, chr8 and chr12.

As noted in Table 1, very few chromosomal abnormalities were observed within the group of benign tumours, which was reflected in the GenoCNA analyses (**Figure 4A**). Discrete gains and losses occurred throughout the genome at low frequencies (<20%), representing CNVs. The most common regions of gains are adjacent to the centromeres on many of the chromosomes, likely indicating repetitive regions. Frequent regions of loss include the HLA region of chr6 (80%), and other common homozygous deletions (as catalogued in **Table S2**).

Discrete CNVs and somatic gains and losses of whole chromosomes and chromosomal arms are reflected in the GenoCNA analyses of the LMP tumours (**Figure 4B**). As expected, chr1 shows loss of the p arm and gain of the q arm in 10–15% of samples, whereas chr12 and chr8 show gains of the entire chromosome. Losses of chr4, chr5, chr6p, chr9p and chr13 are apparent, as are gains of chr7 and chr20 (5–15%).

### Characterization of chromosome 3p12-pcen interval

The ROH in the 3p12-p11 interval, along with the allelic imbalance of chr3 observed in the benign tumour sample 1781T, is interesting in light of recent research in our group suggesting the possibility of tumour suppressor gene(s) in this interval [22], [23], [25]. To investigate this further, we performed mutation analysis in 1781T of protein coding regions and intron/exon splice junction sites of the top 3p12-pcen tumour suppressor gene candidates, *ROBO1*, *GBE1* and *VGLL3* [22], [23], [25]. Several variants, but no apparent deleterious mutations, were observed (**Table 6**).

**Table 6:** Sequencing results of *ROBO1* and *GBE1* in tumour 1781T.

Gene (RefSeq)	Genomic Location	Coding Location	1781T	Ref. NCBI	Ref. Celera	Codon Change	Amino Acid Change	Function	dbSNP	HapMap CEU Frequency
*ROBO1*	g.79067965G>A	c.-610G>A	**A**	G	A			5′UTR	rs1550930	**A = 99.6%**
(NM_133631.3)	g.78796078G>T	c.1346-28G>T	**T**	G	G			intronic	rs2304503	**G = 51.8%**
	g.78737962G>A	c.1892-40G>A	**A**	G	G			intronic	rs967454	**G = 54.9%**
	g.78717508C>T	c.2477-56C>T	**T**	C	C			intronic	rs2255164	**C = 52.2%**
	g.78711350A>G	c.2813-86A>G	**G**	A	A			intronic	rs9864412	**A = 55.3%**
	g.78700779G>T	c.3658+103G>T	**T**	G	T			intronic	rs3925684	T = 97.5%*
	g.78680578A>G	c.4328-123A>G	**G**	A	A			intronic	rs6548592	A = 53.3%*
	g.78676467T>C	c.4721+4T>C	**G**	T	G			intronic	rs7636043	**G = 97.5%**
	g.78676422C>T	c.4721+49C>T	**T**	C	T			intronic	rs7614084	**C = 56.2%**
	g.78666765A>G	c.5128+20A>G	**G**	A	G			intronic	rs9839790	**G = 71%**
	g.78663956C>T	c.5129-6C>T	**T**	C	C			intronic	rs1027832	**C = 55.4%**
*GBE1*	g.81810749C>T	c.-90C>T	**T**	C	C			5′UTR		
(NM_000158.3)	g.81810703delG	c.-44delG	**G**	-	-			5′UTR	rs11391701	**n/a**
	g.81810516G>T	c.143+10G>T	**T**	G	G			intronic	rs9820490	G = 80%*
	g.81720221A>G	c.514-117A>G	**G**	A	A			intronic	rs9863136	**A = 80.6%**
	g.81643167A>G	c.1000A>G	**G**	A	G	ATT>GTT	**Ile>Val**	non-synonymous	rs2172397	**G = 95.9%**
	g.81630214C>T	c.1730+102C>T	**T**	G	G			intronic	rs9870056	**G = 83.9%**
	g.81548210insTTC	c.2335+51insTTC	**insTTC**	-	-			intronic	rs34988523	**n/a**

No sequence variants were observed in *VGLL3*. Genomic locations have been mapped to the February 2009 human reference sequence (GRCh37). An asterisk (*) denotes a CEU Low Coverage panel was used to calculate frequencies as frequency data was not available from the HapMap-CEU population.

BeadChip analysis of 1781T demonstrated extensive allelic imbalance of chr3 and chr9. Chromosome 3 harbours *RASSF1A* (at 3p21.31) and *MLH1* (at 3p22.2), and chr9 harbours *CDKN2A* (9p21.3). These genes have been shown to exhibit tumour suppressor activity, which are often silenced by promoter methylation [35]–[38]. Although the frequency of these events appears to be low in ovarian cancer [35]–[37], we tested the possibility of promoter methylation silencing in the benign tumour case 1781T and our well-characterized EOC cell lines. There was no evidence of promoter methylation of these genes in the analysis of either 1781T-A and 1781T-B, in contrast to evidence of methylated *RASSF1A* alleles in OV-90, TOV-112D, TOV-21G, and TOV-2223G, methylated *CDKN2A* alleles in TOV-112D, and methylated *hMLH1* alleles in TOV-21G (data not shown).

### Characterization of putative homozygous deletion affecting gene function

**Figure 5:**
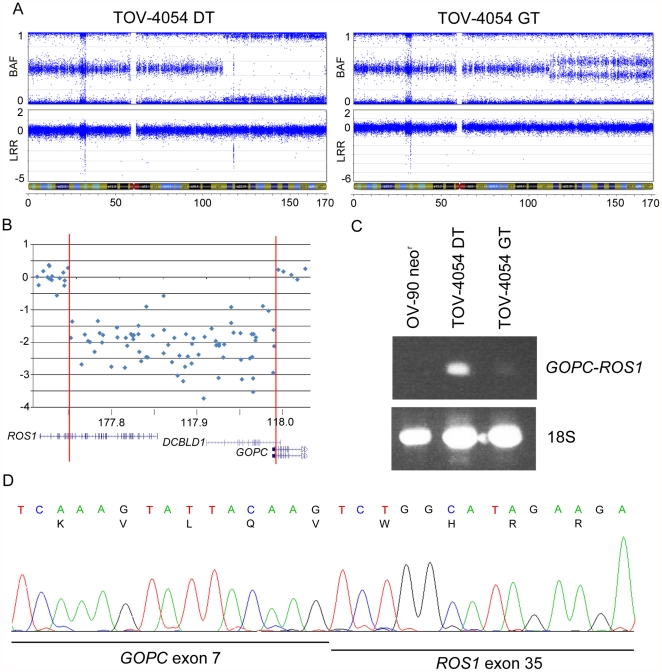
Analysis of the *GOPC-ROS1* fusion gene in an ovarian LMP sample. Analysis of the *GOPC-ROS1* fusion gene. Plots representing the B allele frequency and the Log R ratio on chr6 in TOV-4054GT (left) and TOV-4054DT (right) (**A**). A homozygous deletion is present at 6q22.2 in TOV-4054DT (circled), as observed by a Log R ratio ≤−2 and associated loss of B allele frequency organization. The genomic region located within the 242.5 kb homozygous deletion includes coding exons of the genes *ROS1*, *DCBLD1* and *GOPC*, as visualized by the UCSC Genome Browser (**B**). RT-PCR analysis of the *GOPC-ROS1* fusion gene (**C**). The fusion gene is highly expressed in TOV-4054DT and lowly expressed in TOV-4054GT. The EOC cell line OV-90neo^r^ was used as a negative control. Sequencing of the *GOPC-ROS1* fusion cDNA indicates that exon 7 of *GOPC* is fused in-frame to exon 35 of *ROS1* (**D**).

The inferred 242.5 kb homozygous deletion observed at 6q22.1 in LMP tumour TOV-4054DT stood out in part because it is much larger than the size of the average homozygous deletion (28.3 kb) observed in the present study (**Table 4** **,** **Figure 5A**). The deletion is predicted to affect the function of *ROS1*, *DCBLD1* and *GOPC*, with breakpoints occurring in all three genes (**Figure 5B**). A literature review of these genes reported that *ROS1* and *GOPC* are partners in an oncogenic fusion gene found in the glioblastoma cell line U118MG, created by a 240 kb intrachromosomal deletion. In U118MG, the fusion gene is transcribed from the 5′ end of *GOPC* and contains the first 7 *GOPC* exons and the last 9 *ROS1* exons [39]. Log R ratios indicate that the breakpoints of the 6q22.1 deletion in TOV-4054DT occurred in genomic regions that could possibly result in the creation of an identical fusion gene (**Figure 5B**). To investigate this possibility, we designed an RT-PCR assay to detect the presence of a fusion transcript in cDNA prepared from TOV-4054DT. As shown in **Figure 5C**, TOV-4054DT harbours an aberrant transcript not present in the well-characterized ovarian cancer cell line, OV-90neo^r^, which does not harbour a 6q22.1 anomaly (data not shown). However, a faint band corresponding in size to the aberrant 6q22.1 transcript was also visible in the RT-PCR analysis of the contralateral LMP tumour TOV-4054GT, suggesting a clonal origin of cells that contain this anomaly. This is consistent with observation that both LMP tumours harbor allelic imbalance of the chr6q arm which include the *ROS1*, *DCBLD1*, and *GOPC* loci (**Figure 5A**). Sequence analysis of the aberrant transcript revealed that it was comprised of an in-frame fusion between exon 7 of *GOPC* and exon 35 of *ROS1* (**Figure 5D**). We attempted to detect the fusion protein by Western blot, but the only tissue available for protein extraction was embedded in OCT medium, which was not amenable to further experiments. Interestingly, the fusion transcript is identical to that reported in the U118MG glioblastoma cell line, and to one of the fusion genes identified in a set of cholangiocarcinomas [39], [40]. A review of genotyping data from a minimum of an additional 200 ovarian cancer samples and cell lines of various grades and histopathologies from our laboratory suggest that this chromosomal anomaly is unique to case TOV-4054 (data not shown).

## Discussion

Although LOH of 3p has been reported in benign serous tumours at frequencies of up to 20% [19], [20], loss of 3p alleles were not observed in the analysis of 50 new cases. Interestingly, the sample 1781T, which exhibited 3p14-pcen LOH in our previous LOH study, was shown to exhibit allelic imbalance of chr3 and chr9. Although methylation of *RASSF1A* (3p21.31) and *CDKN2A* (9p21.3) has previously been reported in benign serous tumours at a low frequency [41], [42], we observed no evidence of alteration of promoter CpG methylation in sample 1781T. We also did not detect evidence of promoter methylation of *MLH1* (3p22.2); however, this alteration is more commonly observed in low grade ovarian carcinomas of the endometrioid histopathological subtype [37]. LOH analyses of 3p loci are consistent with SNP analyses suggesting that 3p anomalies are rare occurrences in benign serous tumours, as are anomalies associated with other chromosomes. Although independent LOH analyses have shown low frequencies of loss of chromosomes 6, 7, 9 and 10, only a limited number of loci were examined [43], [44]. Array CGH studies have identified both gains and losses of chr6, and losses of 1p, 4q and 5q [7], [28]. The absence of *KRAS* and *BRAF* mutations in our set of benign tumours is consistent with the paucity of somatic events observed in independent reports [14], [44]. It has been proposed that the acquisition of a *KRAS* or *BRAF* mutation in a benign tumour might initiate the progression to an LMP tumour [4]. The underlying molecular genetic events associated with the development of benign ovarian serous cancer samples remains elusive.

It is possible that an excess of contaminating stromal cells may have obscured chromosomal anomalies in a subset of the samples analyzed. Previous studies using LOH analysis or CGH have observed chromosomal abnormalities without enriching for tumour cells, as chromosomal anomalies present in even 40% of cells can be detected by SNP array analyses [7], [19], [45]. Microdissection of tumour tissues would have necessitated a round of whole genome amplification (WGA), which is discouraged by Illumina. The Illumina Infinium protocol includes a WGA step, and an additional round of WGA has been shown to reduce the call rate and may introduce allelic bias. Hence, it is possible that chromosomal anomalies are underreported in this study. While it was not possible to array constitutional DNA from every patient, a subset of abnormalities observed could be germline CNVs.

It is interesting that the 9.1 Mb ROH at 3p12 observed in sample 1781T overlaps a tumour suppressor region identified by our group using a functional complementation study involving the transfer of chr3 fragments into an EOC cell line [22], [23], and using comparative transcriptome analysis of ovarian serous cancer and normal samples [25]. Although no mutations were identified in 1781T in the targeted analysis of tumour suppressor gene candidates *ROBO1*, *GBE1* and *VGLL3* [22], [23], [25], miRNAs or other noncoding RNAs (ncRNAs), either located within this region or acting upon expression of genes in the region, may play a role in the development of these tumours. Several ncRNAs, predicted to contain miRNA target sites, have been identified in the 3p12.3-pcen interval and shown to be differentially expressed in cancers compared with normal tissues [46]. It is notable that the 3p12 interval was the region of the genome most commonly present in ROHs longer than 5 Mb, and that chr3 harboured both the most number and the longest ROHs (up to 56.6 Mb) of any chromosome within this study. The significance of this observation is unknown but could be influenced by founder effects, as the majority of samples analyzed in our study were from the French Canadian population of Quebec known for its unique genetic demography [33], [34], [47], [48]. A recent genome-wide SNP array analysis of 140 French Canadians from different geographic locations within Quebec reported that subpopulations varied in their genomic structure and degrees of relatedness, and contained significantly more ROHs than samples from European populations [49].

Case BOV-1588 exhibited the most extensive ROHs, as approximately 212 Mb of the genome (7.1%) occurred in ROHs longer than 5 Mb. These ROHs were confirmed to be germline in this patient. As the offspring of first cousins are expected to have about 6.25% genomic autozygosity, it is possible that the extensive ROHs observed in BOV-1588 were the consequence of a consanguineous mating. Upon further review of the medical history of this case, it was revealed that the patient has schizophrenia, a condition that has recently been associated with ROHs [50]. As ROHs may play a role in the etiology of genetic diseases, including cancer [51], further studies are required to determine the significance of these regions in benign ovarian serous tumours.

The chromosomal abnormalities observed in 58 LMP samples from 53 cases mirror those previously reported in the literature, where 1p and 22q are subject to losses, and chr12 and chr8 display increases in copy number [7], [10], [11], [28]–[31], [52]. As expected, *KRAS* and *BRAF* mutations were observed in a mutually exclusive manner [14]. Interestingly, gain of chr12 was significantly associated with the presence of *KRAS* mutations, a finding that has been previously observed [53]. This association was observed in both non-small cell lung cancer (NSCLC) and lung adenocarcinomas, although not in colorectal cancers [54], [55]. Increased *KRAS* expression was observed in NSCLCs harbouring modest increases of copy number of chr12. Another study indicated that NSCLC patients with both a *KRAS* mutation and gain of chr12 had a worse prognosis than those harbouring only one of these aberrations [55]. It would be interesting to investigate this association in LMP cases, but this may be difficult with the low frequency (<15%) and the long average time (>15 years) of recurrences for this disease [3]. To date, only one LMP case, TOV-942GT, has died of cancer, which occurred within a year of the LMP tumour diagnosis; however, the cause of death was pancreatic carcinoma. TOV-942GT harboured an amplification of the *KRAS* locus, and while pancreatic carcinomas have been shown to have a high *KRAS* mutation rate [56], the pathology review excluded the possibility of metastasis in this case. The low frequency of *TP53* mutations in LMP samples is also consistent with independent reports [12], [13]. The *TP53* mutation positive case (TOV-1685GT) was identified in a young patient (age 26), who has remained cancer-free for the follow-up period of 6.5 years. Interestingly, both TOV-1685GT and TOV-942GT harboured extensive evidence of chromosomal instability (CIN) by SNP array analyses. However, low levels of CIN were also observed in a number of *BRAF* and *KRAS* mutation negative cases. Although the relationship between somatic mutations in these genes and genomic anomalies is unknown, the high frequency of CIN in the context of *TP53* mutations combined with the role of p53 in DNA damage response has been proposed in numerous studies (reviewed in Negrini et al., 2010 [57]). Collectively, our results indicate that ovarian serous LMPs are a heterogeneous group, composed of tumours displaying a range of genetic and chromosomal anomalies. It remains to be determined what effects the various anomalies observed in this study have on the clinical presentation of the disease.

The genetic spectrum of abnormalities observed in our small set of LGOSC cases is also consistent with independent reports, particularly when factoring in an independent review of the histopathology of cases. All five LGOSC cases that harboured a somatic *TP53* mutation exhibited extensive CIN and were later reclassified. The overlap in the genetic spectrum of anomalies observed in LGOSC samples with those observed in LMP samples supports the notion that they may share a common molecular genetic etiology. However, the rare instances of *TP53* mutation positive LMP samples (including the LGOSC reclassified as a LMP case) would also support the notion that some LMP samples share common origins with HGOSC as they often exhibit somatic *TP53* mutations and extensive CIN [58]. Regardless of the putative origins of EOC, our results suggest that a combination of *TP53* mutation testing and SNP array analyses may facilitate the classification of malignant serous cases. Identifying methods to improve histopathological classification of serous EOC cases may prove useful as improvements in patient management emerge for treating LGOSC cases.

Few unique homozygous deletions were inferred in the samples analyzed, and none overlapped regions containing known tumour suppressor genes. It is interesting that 28 genes reported as differentially expressed in transcriptome studies of LMP samples are located directly adjacent to or within homozygous deletions identified in our SNP analyses of LMP samples [59], [60]. Furthermore, pairs of differentially expressed genes directly flank six of the observed homozygous deletions. Given the presence of contaminating stromal cells in the samples analyzed, it is likely that many of the homozygous deletions represent germline CNVs, even those found to be unique to a specific case. As CNVs may contain regulatory elements, it is possible that these germline homozygous deletions may affect the expression of adjacent genes, thus contributing to tumour risk or progression (reviewed by Henrichsen et al., [61]). It is also possible that the presence of homozygous deletions may affect chromatin folding, affecting the expression of multiple genes in the region.

A 242.5 kb homozygous deletion at 6q22.1 was observed in the LMP tumour sample TOV-4054DT. Molecular genetic characterization suggests that this resulted in the creation of a transcriptionally active *GOPC-ROS1* fusion gene. To the best of our knowledge, this is the first fusion gene reported in an ovarian LMP context. An identical fusion gene has been described in the glioblastoma cell line U118MG, as well as in a cholangiocarcinoma tumour [39], [40]. Both groups have demonstrated that the GOPC-ROS1 fusion protein is capable of transforming non-malignant cells. This variant protein retains tyrosine kinase activity and is targeted to the Golgi membrane [62]. While it does have oncogenic activity, its aggressivity was augmented when expressed in mice with a disrupted *p16Ink4a* and *p19Arf* locus [63]. Another *GOPC-ROS1* fusion gene was observed in a different cholangiocarcinoma tumour, which resulted in a smaller open reading frame, different cellular location and more potent transforming ability [40]. Although targeted mutation analyses of *ROS1* or *GOPC* have not been performed in cancer samples, the Sanger Wellcome Trust COSMIC database (http://www.sanger.ac.uk/genetics/CGP/cosmic/) reported low frequencies of *ROS1* sequence variations in ovarian (1/84), lung (8/131), breast (2/201), stomach (2/60), colorectal (1/133) and CNS tumours (3/477) [64]. However, a recent large scale exomic genome sequencing analysis of 316 HGOSCs by The Cancer Genome Atlas Research Network identified 5 cases with verified sequence variants. Of the 22 sequence variations observed in either the ovarian TCGA study or in multiple tumour types in the Sanger Wellcome Trust COSMIC database, 17 are missense mutations, with 4 occurring in the tyrosine kinase domain. In total, six mutations have been observed and validated in ovarian tumours, including four missense mutations and two silent mutations [64], [65]. Likewise, one mutation has been observed in *GOPC*; a missense mutation in an ovarian clear cell tumour [64].

The fusion gene occurred in a *TP53*, *KRAS* and *BRAF* mutation-negative context, with evidence of a modest level of CIN in the case sample. The LMP case was bilateral, and although the anomaly was more evident in the right tumour, molecular genetic analysis suggested that both harboured the fusion gene. The clinical and biological significance of this genetic abnormality is not clear. To date, the patient has been cancer free for 1.5 years. However, there is no evidence from a review of SNP array data that it is a common event in LMP, benign or LGOSC samples. Our group is currently investigating SNP array results from HGOSCs and EOC cell lines, and no evidence of a homozygous deletion affecting this region in these aggressive EOC tumours and cell lines were observed (data not shown). It would be interesting to test the effect of the GOPC-ROS1 fusion protein in the context of LMP tumours, but this awaits the development of a suitable cell line model system for this variant of ovarian cancer. Thus we can only speculate based on the effect the identical fusion protein has on the transforming ability in transfected cells, and propose that it may have played a role in the pathology of this LMP tumour [40], [63].

Our results support the hypothesis that LGOSCs are derived from LMP ovarian serous tumours. Interestingly, chromosomal aberrations, but not genetic mutations, were observed in benign serous tumours. It is possible that acquisition of a mutation, such as *KRAS* or *BRAF*, represents the moment of transition from a benign tumour to an LMP. A number of LMP tumours lacking *KRAS* or *BRAF* mutations harboured genomic aberrations, indicating that different initiating events may be present in these tumours. Indeed, a fusion gene known to be oncogenic in other tumour types was found in a single LMP case. While it is unlikely that this fusion gene is a frequent event in the development of LMP tumours, its presence indicates that other initiating, growth-promoting events may be found. The data from this study also indicates that at least some HGOSCs may be derived from LMP tumours. This study also illustrates that there is potential for high-density genotyping arrays in combination with targeted mutation screening to become useful in classifying ovarian serous tumours, and could thus have important implications in management of patients where therapy is targeted based on histopathological subtype.

## Materials and Methods

### Clinical Specimens

Tumour samples and peripheral blood lymphocytes were collected with informed consent from participants undergoing surgeries performed at the Centre hospitalier de l'Université de Montréal-Hôpital Notre-Dame or from surgeries performed at the McGill University Health Centre – Montreal General Hospital. The study is in compliance with the Helsinki declaration, and has been granted ethical approval by the respective Research Ethics Boards of Centre hospitalier de l'Université de Montréal-Hôpital Notre-Dame and The McGill University Health Centre. Clinical features such as disease stage, and tumour characteristics such as grade and histopathological subtype, were assigned by a gynecologist-oncologist and gynecologic-pathologist, respectively, according to the criteria established by the International Federation of Gynecology and Obstetrics (**Table S1**).

### EOC cell lines

EOC cell lines were derived from a stage IIIc/low grade papillary serous adenocarcinoma (TOV-81D), a stage III/high grade clear cell carcinoma (TOV-21G), a stage IIIc/high grade endometrioid carcinoma (TOV-112D), the ascites fluid of a stage IIIc/high grade adenocarcinoma (OV-90), a stage IIIc/high grade serous carcinoma (TOV-2223G), and both the tumour and the ascites fluid of a stage IIIc/high grade serous tumour (TOV-1946 and OV-1946), all from chemotherapy-naïve patients, as described [66], [67]. OV-90neo^r^ is a pSV2NEO-transfected clone of OV-90, which confers resistance to Geneticin® [22].Cells were cultured in OSE Medium supplemented with 2.5 µg/mL amphotericin B, 50 µg/mL gentamicin and 10% FBS as described previously [67].

### Nucleic acid extraction

DNA was extracted from EOC cell lines, fresh frozen tumour specimens and peripheral blood lymphocytes as described previously [68]. For case sample 1781T, non-tumour DNA was extracted from a paraffin-embedded lymph node sample using a previously described method [69].

Total RNA was extracted with TRIzol™ reagent (Invitrogen Canada Inc., Burlington, ON) from the OV-90neo^r^ cell line grown to 80% confluency in 100 mm Petri dishes, or from fresh frozen TOV-4054DT/GT tumours as described previously [70]. RNA quality was assessed by gel electrophoresis or 2100 Bioanalyzer analysis using the RNA 6000 Nano LabChip kit (Agilent Technologies, Mississauga, ON).

### LOH analysis

LOH analysis was performed using polymorphic microsatellite repeat markers representing various 3p loci: *D3S1304* and *D3S1515* at 3p26.2; *D3S1581* and *D3S3640* at 3p21.31; *D3S1274* and *D3S1542* at 3p12.3; *D3S1538* and *D3S2388* at 3p12.2; and *D3S2386* and *D3S2318* at 3p11.2. Genetic analysis of the 3p12 locus in the tumour sample 1781T was determined using seven polymorphic microsatellite markers: *D3S3507*, *D3S1274*, *D3S3049*, *D3S3508*, *D3S3633*, *D3S3679*, *and D3S2318*. The genomic location of the markers was based on February 2009 GRCh37/hg19 assembly of the human reference sequence [71]. LOH analysis was performed using a previously described PCR-based assay, with the primers sets for each marker described in the UniSTS Database (http://www.ncbi.nlm.nih.gov/unists) [21]. LOH or allelic imbalance was scored based on the absence or difference in the relative intensity of alleles in tumour DNA as compared with the DNA from patient-matched peripheral lymphocytes or, in the case of 1781T, DNA from paraffin-embedded lymph node.

### Gene sequencing analysis

Mutation analysis of tumour DNA samples was designed to detect variants in the protein coding exons 2 to 11 of *TP53*, as well as the common mutations in exon 2 of *KRAS* and exons 11 and 15 of *BRAF*. Peripheral blood lymphocyte DNA from case sample TOV-1685GT was also examined for *TP53* mutations in exon 10. Mutation analyses of case sample 1781T were also performed to identify variants in protein coding regions of the chr3 genes *ROBO1*, *GBE1* and *VGLL3*. Mutation analysis was performed using PCR-based assays followed by sequencing of both genomic strands using the 3730XL DNA Analyzer system platform from Applied Biosystems at the McGill University and Genome Quebec Innovation Center (www.genomequebecplatforms.com) as previously described [66], [72]. Primer sequences for each assay were reported previously [23], [72] with alternate primers used for some reactions (Table S3). Primers were designed using Primer3 software based on the genomic structures available from the February 2009 GRCh37/hg19 assembly of the human reference genome. Sequence chromatograms, reviewed by at least two observers, were compared with NCBI reference sequence (RefSeq) reported in GenBank: NM_133631.3 (*ROBO1*), NM_000158.3 (*GBE1*), NM_016206.2 (*VGLL3*), NM_000546.4 (*TP53*), NM_004985.3 (*KRAS*) and NM_004333.4 (*BRAF*). Sequence variants were compared with those reported in the SNP Database (www.ncbi.nlm.nih.gov/SNP). In addition, *TP53* variants were evaluated based on information in the International Agency for Research on Cancer (IARC) TP53 Database (www-p53.iarc.fr).

### Promoter methylation analysis

Promoter hypermethylation of *MLH1*, *RASSF1A* and *CDKN2A* was examined using methylation-specific PCR assays following bisulfite conversion of cytosine residues [73]. The bisulfite conversion reactions were performed using the Imprint™ DNA Modification Kit (Sigma) with 200 ng of DNA from EOC cell lines or tumour tissue. Primer sequences for each assay have been published previously [74]–[76].

### High-density genotyping

Genome-wide chromosomal anomalies in three benign ovarian tumours were inferred using the Infinium™ genotyping technology with Illumina's HumanHap300-Duo Genotyping BeadChip (Illumina, San Diego, CA, USA), which assays >317,500 SNPs. Genotyping of 32 benign ovarian serous tumours (including the 3 tumours assayed on the 300K BeadChip), 58 serous LMP tumours and 12 LGOSCs was performed using Illumina's Human610-Quad Genotyping BeadChip (Illumina, San Diego, CA, USA). This BeadChip assays 620,901 markers, where over 560,000 are SNPs with an average spacing of 4.7 kb per marker (median spacing is 2.7 kb). Both genotyping, using 750 ng of DNA from frozen tumours, and scanning, using the BeadArray™ Reader, were performed at the McGill University and Genome Quebec Innovation Centre (http://gqinnovationcenter.com/index.aspx). All samples had call rates (the percentage of valid genotype calls) within the range of 0.914 and 0.999 (average 0.992). Genotyping results are available at Array Express (in progress).

Genotyping analysis was performed using the Genome Viewer module in BeadStudio Data Analysis software v2.2.22 (Illumina, San Diego, CA, USA.). The software aligns genotyping data for each marker with genomic map coordinates based on March 2006 NCBI36/hg18 (Build 36.1) assembly of the human reference sequence (genome.ucsc.edu/cgi-bin/hgGateway). An image file was created for inferring genomic rearrangements based on the allele frequency and copy number (Log R ratios) for each marker assayed. LOH was inferred by B allele frequency, where values that deviate from 0.5 (less than 0.4 and greater than 0.6) indicate allelic imbalance when reviewed for a series of adjacently mapped markers. Breakpoints were inferred based on deviation of allele frequencies relative to those of adjacently mapped markers. Log R ratios deviating from 0 suggest copy gain or loss. Homozygous deletions were inferred based on Log R ratios ≤−2 for at least three adjacently mapped markers, and sizes were estimated based on the location of nearest flanking markers with Log R ratios above −2. Regions suggesting extensive homozygosity (or runs of homozygosity; ROH), spanning intervals >5 Mb were inferred from heterozygous SNP markers. ROHs were required to have an average frequency of 1 SNP per 10 kb, and a heterozygous call for a marker was allowed if it was flanked by at least 100 SNP markers with homozygous scores [33], [34], [47], [48].

The distribution of mutations in *KRAS*, *BRAF* and *TP53* between the LMP and LGOSC cases was compared using the Fisher Exact test (Statistical Product and Service Solution Package, SPSS, Chicago, IL).

Normalized SNP intensity files were also analyzed by GenoCNA [77]. This software uses a hidden Markov model containing 9 different tumour states, encompassing loss of 1 or 2 copies, copy number neutral LOH, and 5 different gain states allowing for different patterns of allele retention. This model explicitly allows for normal tissue contamination in the samples. Graphs show the percentage of the samples with gains or losses based on the GenoCNA inference, where the percentage is calculated in expectation, using the average of the probabilities of relevant states at each marker.

### Gene expression analysis

Expression of the *GOPC-ROS1* fusion gene was assayed by RT-PCR in TOV-4054DT/GT and OV-90 neo^r^ (negative control) using cDNA synthesized as previously described [78]. Approximately 200 ng of a 1∶10 dilution of the reverse transcribed cDNAs were used in PCR assays. Primers were designed using Primer3 software based on the genomic structures of *GOPC* and *ROS1* and on mRNA sequences available from the February 2009 GRCh37/hg19 assembly of the human reference genome (**Table S3**).

## Supporting Information

Table S1 **Clinical data of ovarian tumour samples.**
(XLS)

Table S2 **Homozygous deletions observed in all samples.**
(XLS)

Table S3 **Novel primers used in the present study.**
(XLS)
